# Nitidine Chloride Alleviates Hypoxic Stress via PINK1-Parkin-Mediated Mitophagy in the Mammary Epithelial Cells of Milk Buffalo

**DOI:** 10.3390/ani14203016

**Published:** 2024-10-18

**Authors:** Zhiwei Kong, Haichang Pan, Zi Wang, Alida Abla, Yingming Wei

**Affiliations:** 1 Guangxi Key Laboratory of Animal Breeding, Disease Control and Prevention, Department of Animal Sciences, Guangxi University, Nanning 530004, China; zhiweikong1987@gxu.edu.cn (Z.K.); haichangpan@163.com (H.P.); wz18846785220@163.com (Z.W.); 13561639940@163.com (A.A.); 2 Institute for Agricultural and Animal Husbandry Industry Development, Guangxi University, Nanning 530004, China

**Keywords:** nitidine chloride, hypoxic stress, mitophagy, PINK1-Parkin pathway, milk buffalo mammary epithelial cells

## Abstract

**Simple Summary:**

Under heat stress, the aerobic metabolic activity of the body is significantly accelerated, which easily leads to hypoxia of high-oxygen consumption tissues, such as the mammary gland. In dairy cattle growing in the high-temperature and high-humidity environment in Guangxi, especially those in the peak period of lactation with strong metabolism, the lack of oxygen in the mammary cells is more obvious, and the milk volume and composition are greatly affected, and even lead to metabolic disorders. Nitidine chloride (NC) is a natural alkaloid with antioxidant properties that can remove excess reactive oxygen species (ROS). However, there is limited information on the effect of NC on BMECs hypoxic injury and its molecular mechanism. In this study, molecular biological methods combined with non-targeted metabolomics were used to study the protective effect of NC on BMECs induced by hypoxic stress. Based on the results obtained, we found that NC has a protective effect on hypoxic mitochondria and regulates amino acid metabolism in response to hypoxic stress.

**Abstract:**

Hypoxia in the mammary gland epithelial cells of milk buffalo (BMECs) can affect milk yield and composition, and it can even cause metabolic diseases. Nitidine chloride (NC) is a natural alkaloid with antioxidant properties that can scavenge excessive reactive oxygen species (ROS). However, the effect of NC on the hypoxic injury of BMECs and its molecular mechanisms are still unknown. Here, an immunofluorescence assay, transmission electron microscopy (TEM), and flow cytometry, combined with untargeted metabolomics, were used to investigate the protective effect of NC on hypoxic stress injury in BMECs. It was found that NC can significantly reduce cell activity (*p* < 0.05) and inhibit cellular oxidative stress (*p* < 0.05) and cell apoptosis (*p* < 0.05). A significant decrease in mitophagy mediated by the PINK1-Parkin pathway was observed after NC pretreatment (*p* < 0.05). In addition, a metabolic pathway enrichment analysis demonstrated that the mechanisms of NC against hypoxic stress may be related to the downregulation of pathways involving aminoacyl tRNA biosynthesis; arginine and proline metabolism; glycine, serine, and threonine metabolism; phenylalanine, tyrosine, and tryptophan biosynthesis; and phenylalanine metabolism. Thus, NC has a protective effect on hypoxic mitochondria, and it can regulate amino acid metabolism in response to hypoxic stress. The present study provides a reference for the application of nitidine chloride to regulate the mammary lactation function of milk buffalo.

## 1. Introduction

Under heat stress, the aerobic metabolic activity of the body significantly accelerates, peripheral vasodilatation increases to regulate body temperature [1], and the blood flow to the rest of the body correspondingly decreases, which can easily lead to hypoxia in tissues with high oxygen consumption, such as the mammary glands [2]. In milk buffaloes growing in a high-temperature and high-humidity environment in Guangxi, especially those in the peak lactation period with vigorous metabolism, hypoxia in the mammary cells is more obvious, and their milk production and composition are greatly affected, even leading to metabolic disorders [3].

Accumulating evidence has shown that hypoxic stress can lead to cellular damage [4], mitochondrial dysfunction [5], oxidative stress [6], and apoptosis [7]. Hypoxic stress is highly correlated with mitochondrial damage [5,8]. In recent years, mitophagy has been found to play an important role in alleviating hypoxic stress [9]. Previous studies have found that hypoxic stress causes mitochondrial damage [10], further leading to the production of large amounts of ROS [11]. Mitophagy can selectively remove damaged and dysfunctional mitochondria to avoid the excessive production of ROS, thus maintaining cellular homeostasis [12]. Yang et al. found that sevoflurane treatment can increase mitophagy by upregulating the HIF-1/BNIP3 signaling pathway to reduce myocardial injury [13]. However, the mechanisms by which mitophagy regulates bovine mammary cell injury under hypoxic stress are unclear.

Nitidine chloride (NC), a natural alkaloid extracted from the root of the Chinese herb pepper, has prominent anti-tumor [14], anti-inflammatory [15], anti-malarial [16], and antioxidant properties [17]. In addition, previous studies found that NC could reduce the occurrence of arrhythmia in myocardial ischemia/reperfusion mice and alleviate myocardial damage caused by ROS [18]. Lin et al. also reported that NC was capable of scavenging ROS to reduce oxidative stress and exert antioxidant activity [17]. However, the majority of studies on NC have mainly focused on its anti-tumor activity, and the effect of NC on hypoxic stress in mammary cells has not yet been reported.

Metabolomics can be used to study the overall and dynamic changes in endogenous small-molecule metabolites [19], and it is a promising approach in the search for potential biomarkers and for the identification of biological pathways in physiological or pathological states [20]. In a previous study, Liao et al. [21] used a metabolomics approach to investigate the protective effects of the plant active ingredient salidroside on hypoxic cells and their main targets [22]. In this study, we hypothesized that NC could alleviate hypoxic stress in the mammary gland cells of dairy buffaloes by increasing mitochondrial autophagy. To verify our hypothesis, an in vitro hypoxic stress model of BMECs was constructed, and an UHPLC-OE-MS-based metabolomics analysis was performed to investigate the metabolomic perturbations and protective effects of NC in hypoxic BMECs. This study is of great significance for understanding the mechanisms of nutrient regulation under hypoxia.

## 2. Materials and Methods

### 2.1. Cell Culture and Treatment

BMECs were isolated and cultured by following previously described methods [23]. Briefly, we selected a 12 gauge biopsy needle to collect appropriate tissue from the midpoint of the upper quarter of the posterior udder of lactating buffalo at the late age of 2 years and immediately transported it to the laboratory. After washing with DPBS (D8662, Sigma-Aldrich, St. Louis, MO, USA), the tissue was cut into small pieces of 1 mm^3^. Milk buffalo mammary epithelial cells with high purity were obtained by using the tissue block method. The purified cells were incubated in DMEM/F-12 (R8758, Sigma-Aldrich, St. Louis, MO, USA) containing 10% FBS (10099141, Gibco, Carlsbad, CA, USA) and 1% antibiotics (penicillin/streptomycin; Invitrogen, Carlsbad, CA, USA) at 37 °C, 5% CO_2_, and saturated humidity in an incubator (DH-160I, SANTN, Shanghai, China). The logarithmically grown cells were taken and spread on a plate inside a 10 cm medium dish. After most cells attached to the bottom wall of the culture dish, they were grouped as follows: the cells left untreated (21% O_2_) formed the control group; the cells incubated for 72 h in a hypoxic (1% O_2_) environment formed the hypoxia group; and the cells incubated for 72 h in a hypoxic environment (1% O_2_) after 2 h of pretreatment with 25 μM nitidine chloride (purity ≥ 98%, DL0055-0020, Desite, Chengdu, China) formed the NC group. The medium was changed every 2 or 3 days.

### 2.2. EDU Detection

To determine cell proliferation after hypoxia and NC treatment, a 5-ethyl-2′-deoxyuracil (EdU) staining kit was used. In brief, the steps were as follows: First, 10 μM EdU was incorporated into the treated cells, and, after 1 h, the cells were fixed in 4% paraformaldehyde. The cells were then extracted with 0.3% Triton X-100 for 15 min and washed with PBS. Next, the cells were incubated at room temperature with the Click-iT reaction mixture for 30 min and treated with 5 mg/mL 4′,6-diamino-reverse staining 2-phenylindoles (DAPI). Finally, the treated cells were photographed with an inverted fluorescence microscope (Axio Observer D1, ZEISS, Oberkochen, Germany), and the number of positive cells was counted.

### 2.3. Immunofluorescence

Cell crawls with BMECs were prepared and fixed with 4% paraformaldehyde for 30 min, rinsed with PBS (AWC0409, Abiowell, Changsha, China) for 5 min × 3 times, and permeabilized with PBS containing 0.3% Triton X-100 (AWB0141, Abiowell, Changsha, China) for 30 min at room temperature. Subsequently, the crawls were closed with 5% BSA (Saibao, Yancheng, China) for 60 min at room temperature, appropriately diluted primary antibodies (LC3 and TOM20; detailed information is shown in Table S1) were added dropwise, and the crawls were incubated at 4 °C overnight. After washing 3 times with PBS, the cells were incubated with the relevant secondary antibodies (Table S2) for 60 min at 37 °C and rinsed 3 times with PBS. The nuclei were stained with DAPI (AWC0291, Abiowell, Changsha, China) at 37 °C for 10 min, washed 3 times with PBS, and blocked with buffered glycerol. Finally, the cell culture slides were observed under a fluorescence microscope (BX50, Olympus, Tokyo, Japan).

### 2.4. Cell Apoptosis Detection

The cells treated with hypoxia and NC were first digested and collected with a pancreatic enzyme without EDTA, and the cell concentration was not less than 1 × 10^5^. Then, a 500 uL binding buffer was added to suspend the cells, and Annexin V-APC (KGA1030, Kaiji, Nanjing, China) and propidium iodide (PI) were mixed evenly and added to the cell suspension. A light-resistant reaction was carried out at room temperature. Finally, the apoptosis rate was observed and recorded via flow cytometry (BD FACScalibur, San Jose, CA, USA).

### 2.5. Detection of Cell Oxidative Damage Indices

After the hypoxia and NC treatment, the change in the reactive oxygen species (ROS) fluorescence intensity in each group was detected via flow cytometry. The levels of antioxidant-related enzymes, such as superoxide dismutase (SOD) (Item No. A001-3), malondialdehyde (MDA) (Item No. A003-1), catalase (CAT) (Item No. A007-1-1), glutathione peroxidase (GSH-Px) (Item No. A005-1), and total antioxidant capacity (T-AOC) (Item No. A015-1), were measured according to the kit’s instructions. All kits were purchased from the Nanjing Jiancheng Bioengineering Institute (Nanjing, China).

### 2.6. Transmission Electron Microscopy (TEM) Analysis

The treated cells were fixed with 2.5% glutaraldehyde and 1% osmic acid, and then they were dehydrated with ethanol solution, uranoxy acetate, and propylene oxide at different concentration gradients. Next, the cells were soaked in propylene oxide and epoxy resin at a concentration ratio of 1:1, and then they were embedded in epoxy resin, cut into ultra-thin sections using a microtome, and dyed with lead and uranium. Finally, images were taken and recorded via transmission electron microscopy (JEM-2000EX, JEOL Co., Tokyo, Japan).

### 2.7. Protein Separation and Western Blot Analysis

Referring to a previous method [24], the total protein of the BEMCs was extracted with a RIPA buffer (AWB0136, Abiowell, Changsha, China), and the protein concentration was determined by using a BCA kit (AWB0104, Abiowell, Changsha, China). The protein supernatant was mixed with loading buffer (AWB0055, Abiowell, Changsha, China), incubated in boiling water for 5 min, and then placed in an ice box for spare. Protein samples were separated via SDS-PAGE, and then the proteins were transferred to a polyvinylidene fluoride (PVDF) membrane. The membrane was transferred to a 5% BSA blocking solution, blocked for 90 min at room temperature, and incubated with primary antibodies (β-actin, Drp1, VDAC1, Parkin, PINK1, Mfn1, Mfn2, and LC3B; detailed information is shown in Table S1) overnight at 4 °C. After washing the membrane 3 times with 1 × Tris-buffered saline (AWB0074, Abiowell, Changsha, China), it was co-incubated with the corresponding secondary antibodies (Table S2) at room temperature for 90 min. Images were taken and analyzed by using an Alpha Imager 2200 digital imaging system (Digital Imaging System, Kirchheim, Germany).

### 2.8. Metabolomics

#### 2.8.1. Sample Preparation

For sample preparation, 600 μL of pre-cooled (−40 °C) methanol (containing isotope-labeled internal standard mixture) was added to the BEMC samples, vortexed and mixed for 30 s, sonicated for 10 min in an ice-water bath, and left to stand at −40 °C for 1 h. The samples were centrifuged at 12,000 rpm and 4 °C for 15 min. The supernatant was transferred to injection vials, and an aliquot of the mixed quality control (QC) samples in the injection vials was taken for on-board detection.

#### 2.8.2. UHPLC-OE-MS Analysis

An ultra-high-performance liquid chromatography (UHPLC) system (1290, Agilent Technologies, Santa Clara, CA, USA) combined with Q Exactive (Orbitrap MS, Thermo Fisher Scientific, Waltham, MA, USA) was used for a liquid chromatography–mass spectrometry/mass spectrometry (LC-MS/MS) analysis. Mobile phase A was used in the instrument and was composed of a 0.1% formic acid aqueous solution, which acted as a positive electrode. Mobile phase B was acetonitrile, and the aqueous ammonium acetate solution was negative. The sample volume required for testing was 3 μL. The MS/MS spectra were obtained on an information-dependent basis (IDA).

For data processing in metabolomics, the original data were converted to mzXML format using proteowizard and processed using an in-house program. The program was developed by R and is based on xcms for peak detection, extraction, comparison, and integration. Metabolite annotation was then performed using the MS2 internal database (BiotreeDB, V2.1). The cut-off value for annotation was set to 0.3.

### 2.9. Statistical Analysis

SPSS 23.0 software (IBM Corporation, Armonk, NY, USA) was mainly used for the data analysis. The immunofluorescence intensity and relative expression of the mitochondrial autophagy pathway proteins in the cells were analyzed using a one-way ANOVA. *p <* 0.05 was considered significant, with being *p <* 0.01 extremely significant, and the data are displayed as mean ± SD.

A principal component analysis was performed on the normalized raw data to observe their reliability. An orthogonal partial least squares discriminant analysis (OPLS-DA) was used to filter out ineligible metabolites. A univariate statistical analysis (UVA) was used to filter differential metabolites [*p <* 0.05, variable importance projection (VIP) > 1] and to plot volcano diagrams. KEGG annotation of differential metabolites were based on the Kyoto Encyclopedia of Genes and Genomes (KEGG) pathway database and PubChem. All pathways of differential metabolite mapping of the corresponding species Bos taurus (cow) were sorted out.

## 3. Results

### 3.1. NC Alleviates Hypoxia-Induced Cellular Damage in BMECs

**Figure 1:**
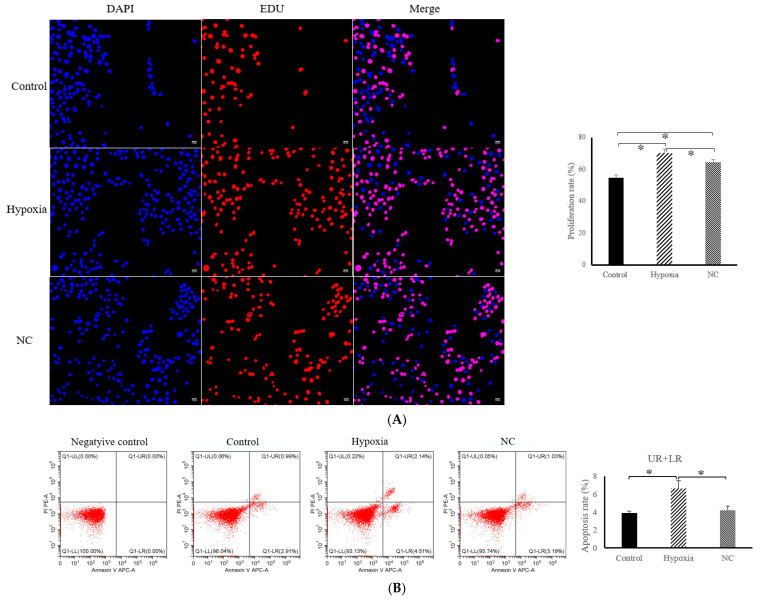
Changes in cell proliferation and apoptosis. (**A**) EDU proliferation assay was used to detect proliferating on each group of cells. Scale bar = 50 um. Blue represents the staining of the nucleus and red represents the staining of the DNA. (**B**) Apoptosis rate under hypoxia and NC conditions detected via flow cytometer. * *p* < 0.05, represents a significant difference.

The changes in cell proliferation and apoptosis after the hypoxia and NC treatment are shown in Figure 1. Compared with the control group, the EDU-positive cell rate in the hypoxia group significantly increased (*p* < 0.05), while the EDU-positive cell rate in the NC group significantly decreased (*p* < 0.05) compared with the hypoxia group (as shown in Figure 1A). Correspondingly, after the hypoxia treatment, the apoptosis rate of the hypoxia group was significantly higher than that of the control group (*p* < 0.05). In contrast, the apoptosis rate of the NC-treated cells was significantly lower than that of the hypoxia group (*p* < 0.05) (Figure 1B).

### 3.2. NC Reduces Oxidative Stress Event of Hypoxia-Induced BMECs Cells Injury

**Figure 2:**
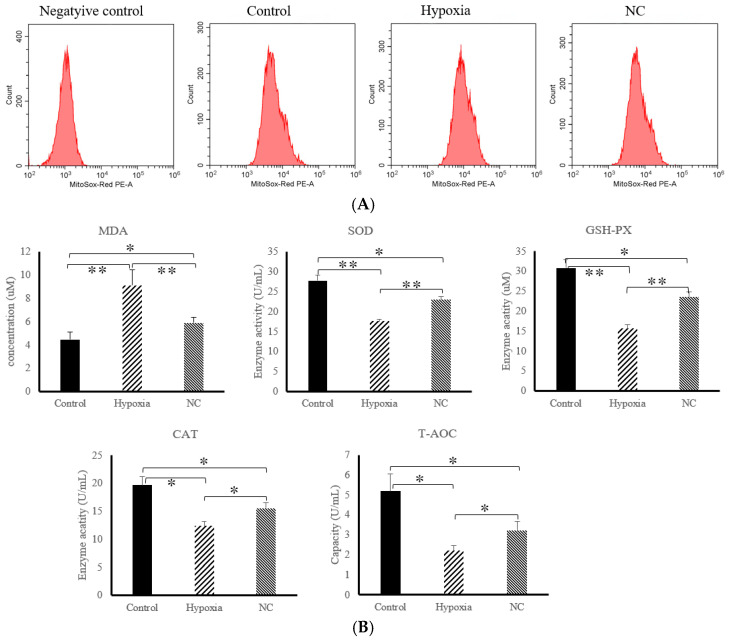
The anti-oxidative stress effects of NC on hypoxia-induced BMEC cell injury. (**A**) Fluorescence intensity of ROS under hypoxia and NC conditions detected via flow cytometry. (**B**) Effects on MDA, SOD, GSH-PX, CAT, and T-AOC levels in BMECs under hypoxia and NC conditions. * = *p*. * *p* < 0.05, represents a significant difference; ** *p* < 0.01, represents an extremely significant difference.

Figure 2A shows the fluorescence intensity of mitochondrial reactive oxygen species (ROS) detected via flow cytometry. The results show that, compared with the control group, the fluorescence intensity of ROS in the hypoxia group significantly increased (*p* < 0.05), and the intracellular fluorescence intensity of ROS in the NC group was significantly lower than that in the hypoxia group (*p* < 0.05). Figure 2B shows the results of the antioxidant-related indicators. Compared with the control group, the MDA level in the hypoxia group significantly increased (*p* < 0.05), while the activities of SOD, GSH-PX, CAT, and T-AOC significantly decreased (*p* < 0.05); furthermore, the MDA level in the NC group significantly decreased (*p* < 0.05), while the activities of SOD, GSH-PX, CAT, and T-AOC significantly increased (*p* < 0.05). In addition, in the NC group, the intracellular MDA level was significantly lower than that in the hypoxia group (*p* < 0.05), while the activities of SOD, GSH-PX, CAT, and T-AOC were significantly higher than those in the hypoxia group (*p* < 0.05).

### 3.3. NC Alleviates Hypoxia-Induced Mitophagy in BMECs

**Figure 3:**
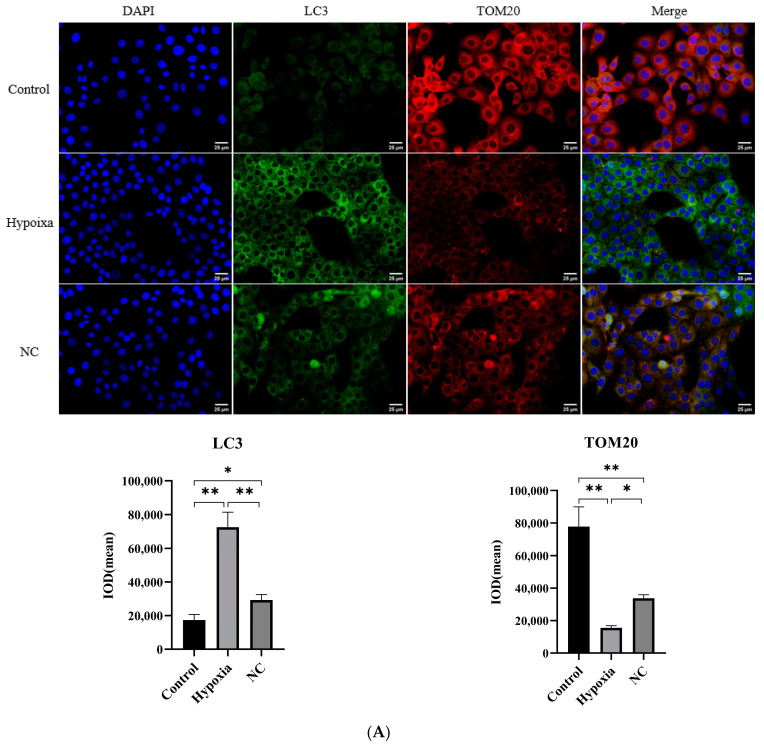

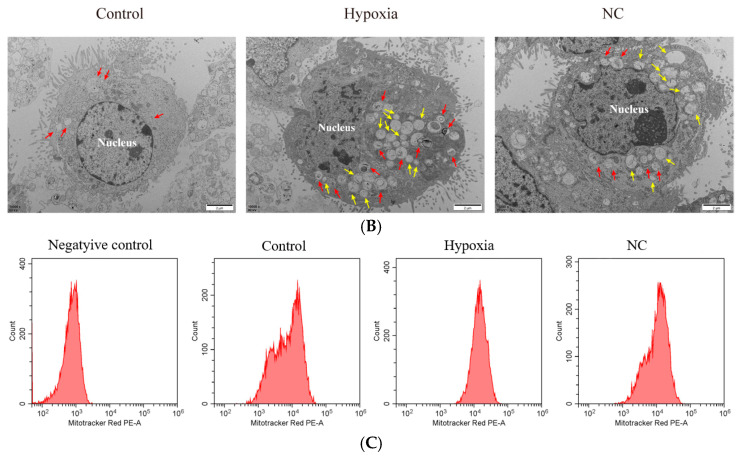
Effect of NC on mitochondrial structure and function in BMECs. (**A**) Fluorescence intensity of LC3 and TOM20 under hypoxia and NC conditions detected via immunofluorescence. (**B**) Changes in mitochondrial ultrastructure of BMEC s under hypoxia and NC conditions. (**C**) Fluorescence intensity of mitochondrial function under hypoxia and NC conditions detected via flow cytometry. (*n* = 3, * *p <* 0.05 means significant, ** *p <* 0.01 means extremely significant) Note: NC, nitidine chloride. The red arrows in this figure are autophagic vesicles or autophagic lysosomes, and the yellow arrows are vacuolated structures. Note: NC, nitidine chloride.

We used fluorescence microscopy to observe the localization of the autophagy marker LC3B and the mitochondrial membrane protein TOM20 (Figure 3A). It was found that the fluorescence intensity of LC3B in the hypoxia and NC groups was significantly enhanced (*p* < 0.05) compared with that in the control group, and that in the NC group was significantly decreased compared with that in the hypoxia group (*p* < 0.05). The fluorescence intensity of TOM20 showed the opposite trend to LC3B under the same treatment conditions. The effects of NC on the ultrastructure of the BMECs are shown in Figure 3B. The results show that the BMECs in the control group had better morphology and cellular integrity (Figure 1). In contrast, the cell membrane of the hypoxia group was ruptured, with many inclusions spilling out, and the whole morphology of the cells was incomplete. The cell morphology of the NC group was better than that of the hypoxia group, with a small portion of cells having an incomplete morphology. The difference in autophagic vesicles among the three groups was not significant, with the hypoxia group having slightly more than that of the control and NC groups. Figure 3C shows the results of flow cytometry on Mitotracker Red-labeled mitochondrial function. The results show that, compared with the control group, both the hypoxia and NC treatment significantly improved the fluorescence intensity of mitochondrial function (*p* < 0.05), and the intracellular fluorescence intensity of the NC group was significantly lower than that of the hypoxia group (*p* < 0.05).

### 3.4. NC Alleviates Mitophagy by Reducing Activation of the PINK1-Parkin Pathway

**Figure 4:**
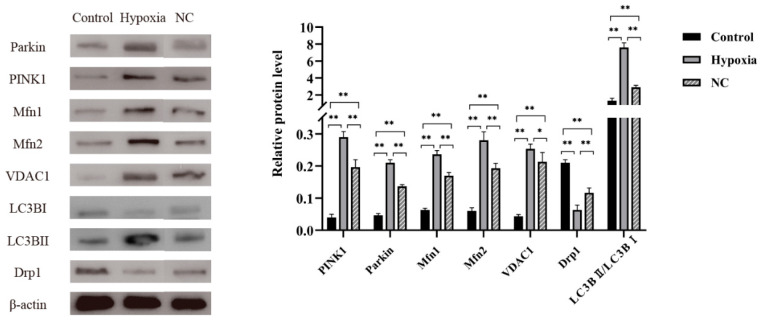
Protein expression of the mitophagy pathway-related receptors. (*n* = 3, * *p <* 0.05 means significant, ** *p <* 0.01 means extremely significant).

In this study, we further investigated the mechanism by which NC alleviates hypoxia-induced mitophagy in BMECs. Western blot was used to examine the expression of the mitophagy pathway-related proteins PINK1, Parkin, Mfn1, Mfn2, VDAC1, Drp1, and LC3B in the cells. The Western blot results showed that the expression levels of PINK1, Parkin, Mfn1, Mfn2, VDAC1, and LC3BII/LC3BⅠ were significantly higher in the hypoxia and NC groups (*p* < 0.05) than in the control group, while the expression level of Drp1 was lower (*p* < 0.05) in the hypoxia and NC groups than in the control group (Figure 4). After pretreatment with NC, the expression levels of PINK1, Parkin, Mfn1, Mfn2, VDAC1, and LC3BII/LC3BⅠ were significantly lower in the NC group (*p* < 0.05) than in the hypoxia group, while the expression level of Drp1 was higher (*p* < 0.05) in the NC group than in the hypoxia group (Figure 4).

### 3.5. UHPLC-OE-MS Untargeted Metabolomics Analysis

**Figure 5:**
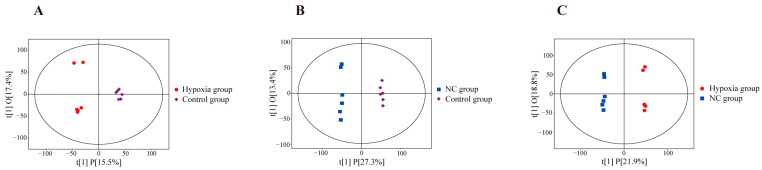
Graph of OPLS-DA scores. (**A**) Differences between hypoxic and control groups. (**B**) Differences between NC and control groups. (**C**) Differences between hypoxic and NC groups.

In order to reveal the effects of NC on the cellular metabolism of BMECs, we analyzed the metabolites based on LC-MS to clarify the metabolomic differences between the control, hypoxia, and NC groups. After data normalization, an orthogonal partial least squares discriminant analysis (OPLS-DA) was first performed to outline the inter-group differences in metabolites. The OPLS-DA score plot showed a clear separation between the groups (Figure 5). The R^2^Y value in the OPLS-DA model for the hypoxia and control groups was 0.985, and the Q^2^ value was 0.4. The R^2^Y and Q^2^ values for the NC and control groups were 0.998 and 0.915, respectively. Additionally, the R^2^Y and Q^2^ values for the hypoxia and NC groups were 0.999 and 0.872, respectively, showing high applicability and predictability. The OPLS-DA model was not overfitted and was more reliable.

**Figure 6:**
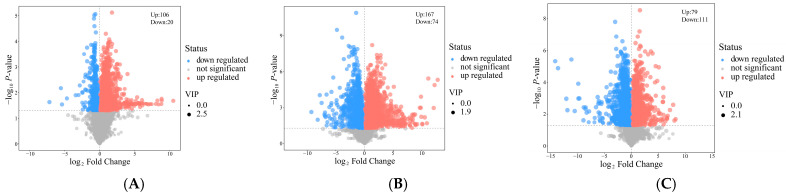

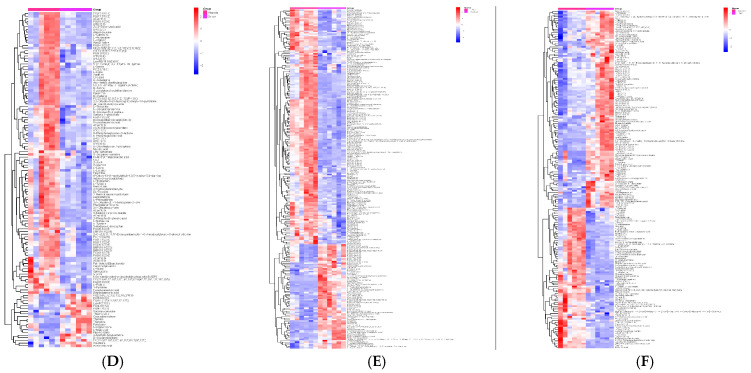
(**A**–**C**): Volcano plot of differential metabolite screening. Scatter size corresponds to VIP value size. Red indicates significantly upregulated metabolites, blue indicates significantly downregulated metabolites, and gray indicates non-significantly different metabolites. (**A**) Comparison of hypoxia and control groups. (**B**) Comparison of NC and control groups. (**C**) Comparison of hypoxia and NC groups. (**D**–**F**) Heatmap visualization of metabolomic alterations induced by NC. Red indicates high expression of the substance content, and blue indicates low expression of the substance content. (**D**) Comparison of hypoxia and control groups. (**E**) Comparison of NC and control groups. (**F**) Comparison of hypoxia and NC groups.

VIP values were generated based on the OPLS-DA model. Subsequently, screening was conducted to identify differential metabolites that contributed significantly to the separation between the groups based on VIP > 1, while Student’s *t*-test was used to further restrict these differential metabolites (*p <* 0.05). Compared with the control group, a total of 126 and 241 significant differential metabolites were identified in the hypoxia and NC groups, respectively (VIP > 1, *p <* 0.05). Additionally, 190 significant differential metabolites were identified in the NC group compared with the hypoxia group (VIP > 1, *p <* 0.05). Observations of volcano plots indicated that the NC treatment significantly altered the metabolic activities of the BMECs, and the NC treatment on BMECs affected more metabolites than did the hypoxia treatment (Figure 6A–C). Heat maps constructed from 45, 130, and 76 different metabolites (VIP > 1, *p <* 0.05, and fold change > 2) were used to visualize the relative levels of the differential metabolites for each sample, and the differential metabolites were grouped clearly and reproducibly (Figure 6D–F).

**Figure 7:**
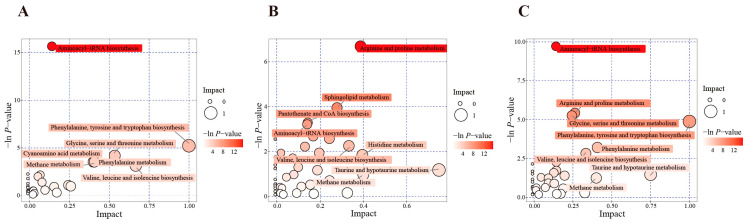
Plot of pathway analysis. Horizontal coordinates and bubble sizes indicate the magnitude of the influencing factor of the pathway. Vertical coordinates and bubble colors indicate the *p*-value (−ln(*p*)) of the enrichment analysis. The darker the color, the smaller the *p*-value and the more significant the enrichment. (**A**) Comparison of hypoxia and control groups. (**B**) Comparison of NC and control groups. (**C**) Comparison of hypoxia and NC groups.

We then used the Kyoto Encyclopedia of Genes and Genomes (KEGG) database for a further enrichment analysis of differential metabolite pathways to identify the key pathways with the highest correlation with metabolite differences. The metabolic pathway analysis results showed that, compared with the differential metabolites in the control group, those in the hypoxia group were mainly enriched in aminoacyl tRNA biosynthesis; phenylalanine, tyrosine, and tryptophan biosynthesis; glycine, serine, and threonine metabolism; and valine, leucine, and isoleucine biosynthesis (Figure 7A). The differential metabolites in the NC group were mainly enriched in arginine and proline metabolism, sphingolipid metabolism, pantothenic acid and CoA biosynthesis, and aminoacyl tRNA biosynthesis (Figure 7B). Compared with the differential metabolites in the hypoxia group, those in the NC group were mainly enriched in aminoacyl tRNA biosynthesis; arginine and proline metabolism; glycine, serine, and threonine metabolism; phenylalanine, tyrosine, and tryptophan biosynthesis; and phenylalanine metabolism (Figure 7C).

Metabolites that could be used to explain the differences between the control group and the hypoxia and NC groups were screened according to VIP > 1 and *p <* 0.05. Compared with the control and NC groups, the following had a higher abundance in the hypoxia group: L-phenylalanine, L-tyrosine, L-leucine, L-lysine, L-threonine, L-arginine, L-serine, and L-tryptophan in the aminoacyl tRNA biosynthesis pathway; L-arginine, creatine, and phosphocreatine in the arginine and proline metabolism pathway; L-serine, L-threonine, D-serine, and creatine in the glycine, serine, and threonine metabolism pathway; L-phenylalanine and L-tyrosine in the phenylalanine, tyrosine, and tryptophan biosynthesis pathways; and dihydrosphingosine and L-serine in the sphingolipid metabolism pathway (Table 1). Putrescine in the arginine and proline metabolism pathways had a lower abundance in the hypoxia group than in the control and NC groups (Table 1). Compared with the control and hypoxia groups, the following had a higher abundance in the NC group: L-proline and L-valine in the aminoacyl tRNA biosynthesis pathway; L-glutamine and L-proline in the arginine and proline metabolism pathway; sphingomyelin in the sphingolipid metabolism pathway; and L-valine, dephosphorylated CoA, and uracil in the pantothenic acid and CoA biosynthesis pathway (Table 1).

## 4. Discussion

In animals, hypoxic stress first attacks the mitochondria, leading to mitochondrial dysfunction, which triggers cellular metabolic disorders [24]. It has been reported that NC has antioxidant activity capable of scavenging excess ROS [17], but the effect of NC on hypoxic stress in animals and its underlying mechanisms are unknown. In the present study, a UHPLC-OE-MS-based metabolomics analysis, combined with EDU staining, immunofluorescence detection, transmission electron microscopy, flow cytometry, and Western blot assays, was used to investigate the effects of NC on hypoxic stress and its potential mechanisms. We found a better cell morphology and a reduction in oxidative damage and mitophagy mediated by the PINK-Parkin pathway of BMECs in the NC-treated group, thus providing evidence that NC can alleviate hypoxic stress.

When exposed to hypoxia, cells undergo a variety of pathological reactions that can cause cell damage, including apoptosis, oxidative stress, and mitochondrial dysfunction [23]. In this study, we found that hypoxia improved cell viability and mitophagy, which might be due to the important role of mitophagy in cell protection, where misfolded proteins and damaged organelles are degraded under hypoxic stress [25]. This is also confirmed by the changes in mitochondrial membrane integrity and mitochondrial ultrastructure observed in the experimental results. In addition, hypoxia increased MDA levels and ROS production, and it reduced SOD, CAT, GSH-PX, and T-AOC activities; these results are consistent with those of previous research [26]. This may be because, in the case of hypoxia, the supply of electron-acceptor oxygen molecules at the end of the electron transport chain is insufficient, resulting in a large number of ROS [26]. Studies have indicated that hypoxia is often associated with apoptosis and that ROS can promote apoptosis in response to hypoxic stress injury [27], and this was verified by the increase in intracellular apoptosis and ROS production observed in the hypoxia group in this study.

Studies have shown that NC can inhibit the proliferation of tumor cells, which is consistent with the results of this study; this is possibly because NC can inhibit the mTORC1 signaling pathway [28], which is capable of regulating cell proliferation and autophagy [29]. In this study, we also found that the addition of NC alleviated the oxidative damage caused by hypoxia, which is consistent with the findings of Baskaran et al. [30]; this is possibly because NC can regulate mitochondrial function and structure to relieve the oxidative stress caused by hypoxia. This was also confirmed by the changes in mitochondrial structure and function observed in this experiment. Studies have shown that NC can induce the apoptosis of ovarian cancer cells through the Akt/mTOR pathway [31], which is inconsistent with the results of this study. In the present work, we found that NC reduced apoptosis, possibly because alkaloids can alleviate apoptosis by reducing the expression of caspase-3. Apoptosis and autophagy often coexist. Mitophagy, as a defense mechanism, can remove damaged mitochondria and excess ROS and promote cell survival in stressful environments [32]. In this study, by detecting the autophagy marker LC-3B and the mitochondrial membrane integrity marker TOM20 and observing the mitochondrial ultrastructure, we found that hypoxia could induce autophagy in BMECs. Supplementation with NC reduced the conversion of LC-3BI to LC-3BII, improved mitochondrial membrane integrity, and reduced the number of autophagosomes, possibly due to NC maintaining cell survival by inhibiting mitophagy [33].

Mitophagy is an important mitochondrial quality control mechanism that removes damaged mitochondria and reduces ROS production [34]. The activation of mitophagy promotes the fusion of damaged mitochondria and reduces excessive mitochondrial fragmentation [35], thus removing dysfunctional mitochondria, thereby reducing ROS production and protecting cells from damage under environmental stress [36]. This is inconsistent with the changes in cell activity, ROS, and mitochondrial function found in this study, which might due to the fact that excessive mitophagy reduced cell viability [37], elevated ROS levels, and aggravated cell damage [38] and death. The diversity of the relationship between mitophagy and various cellular injuries may be related to the inconsistent role played by mitophagy in different disease models [39]. In the present study, we found that NC was able to alleviate hypoxia-induced mitophagy and protect the integrity of BMECs by inhibiting the PINK1-Parkin pathway. Our findings are consistent with those of previous studies [40,41] that found that the inhibition of excessive mitophagy helps to prevent cell death. This may be attributed to the fact that, on the one hand, the treatment of cells with NC, as an antioxidant, led to a reduction in the ROS produced by the cells in the initial stage of hypoxia, which decreased the damage to mitochondria. In this case, the mitochondria were able to perform their normal functions and did not need to activate excessive mitophagy as a defense mechanism [17]. On the other hand, mitochondrial dynamics were balanced and functionally intact, and the mitochondria produced a certain amount of ROS to avoid excessive cellular damage [42].

As centers of cellular metabolic activity, mitochondria rely on their own highly ordered ultrastructure to maintain orderly metabolic activity [43]. Therefore, mitochondrial dysfunction can lead to metabolic disorders. In order to study the changes in the metabolites in the mammary epithelial cells after the hypoxia and NC treatment, we performed a non-targeted metabolomics analysis. We found that the pathways with significant changes after the NC treatment were mainly enriched in aminoacyl tRNA biosynthesis; arginine and proline metabolism; glycine, serine, and threonine metabolism; phenylalanine, tyrosine, and tryptophan biosynthesis; and phenylalanine metabolism. The alterations in these pathways reflect the adaptation of BMECs to hypoxic stress and the potential mechanisms by which NC can alleviate such stress.

Aminoacyl-tRNA biosynthesis is essential for the biosynthesis of proteins critical for cellular function [44]. Our findings show that differential metabolites, such as L-phenylalanine, L-tyrosine, L-leucine, L-threonine, L-serine, L-tryptophan, and L-valine, were significantly upregulated in the aminoacyl-tRNA biosynthesis pathway during hypoxia. It has previously been reported that hypoxia exposure inhibits energy metabolism, leading to amino acid and nucleotide deficiencies [45]. Amino acids associated with glycolysis in organisms under hypoxic stress, including the phosphoglycerate family (serine), shikimate family (tyrosine), and pyruvate family (alanine, leucine, and valine), are significantly elevated [46], which is consistent with our findings. In addition, the BMECs pretreated with NC downregulated amino acids such as L-leucine and L-lysine that were upregulated due to hypoxia. In general, leucine may improve cellular stress and anti-inflammatory responses by regulating cellular metabolic processes [47]; however, a previous study suggests that high leucine levels interfere with the metabolism of other macro-neutral amino acids, are neurotoxic, and can also lead to oxidative stress [48] in animals, which may support our findings. Lysine has antioxidant properties, and several studies have shown that hypoxia can lead to an increase in lysine levels, thereby improving overall antioxidant capacity [49], which is consistent with the findings of our study. This may indicate that NC exerts its antioxidant activity by replacing some of the lysine in its antioxidant function. However, L-proline and L-valine levels were significantly upregulated by hypoxia after NC pretreatment compared with only 72 h of hypoxia, which may be related to the regulation of energy metabolism and the organism’s own antioxidant activity induced by NC [50]. Moreover, previous studies have pointed out that enhanced proline metabolism under hypoxia leads to a decrease in intracellular ROS levels [51] and that enhanced proline metabolism promotes the tricarboxylic acid cycle and glutathione biosynthesis, eliminates ROS, maintains cellular energy under stress conditions, and is able to maintain membrane integrity and cellular osmotic homeostasis [52]. This further suggests that NC has the potential to regulate amino acid metabolism and thus influence redox reactions.

Previous studies have indicated that amino acids, such as threonine and serine, accumulate under hypoxic stress [53], and that the upregulation of the glycine, serine, and threonine metabolic pathways inhibits the hypoxia-inducible factor signaling pathway, thereby decreasing intracellular ROS levels [54]. This study supports our results. In our study, NC pretreatment significantly downregulated the differential metabolites of the glycine, serine, and threonine metabolic pathways compared with the hypoxia treatment, showing a trend of reversal toward the control group. This may be due to the fact that NC modulates intracellular ROS production [17], replacing some antioxidants upregulated by hypoxic stress and reducing the level of metabolite perturbation.

In addition, the phenylalanine, tyrosine, and tryptophan biosynthesis pathways, as well as the phenylalanine metabolic pathways, showed the same trend and were significantly downregulated in the BMECs pretreated with NC followed by hypoxia. These two pathways play an important role in hypoxic stress [55]. Phenylalanine is an important ketogenic amino acid, and it has been suggested that the biosynthesis of phenylalanine, tyrosine, and tryptophan increases after hypoxic stress to supplement the deficient energy supply [56], which is consistent with our study. However, phenylalanine is also a precursor of amino acids for the formation of neurotransmitters (e.g., dopamine) [57], and high concentrations of phenylalanine and tyrosine are neurotoxic and can cause neurological damage [58]. Therefore, for animals, NC treatment is important for regulating energy supply and cell permeation and preventing cell damage caused by hypoxic stress.

In this study, L-arginine, creatine, and phosphocreatine in the arginine and proline metabolic pathways were significantly upregulated and putrescine was significantly downregulated under hypoxic stress, whereas NC pretreatment was able to reverse this phenomenon and significantly upregulated L-glutamine levels. Previous studies support our results [59]. It has been shown that increased arginine levels can enhance antioxidant responses by producing more nitric oxide to reduce hypoxic injury [53,59]. Glutamate and putrescine are significantly reduced in hypoxia [60], and glutamate is consumed in large amounts, allowing for the activation of the metabolic pathways of arginine and proline, which are converted to replenish glutamate [61]. Glutamate synthesizes not only glutathione, but also glycine and cysteine, all of which are associated with antioxidant function [61]. Thus, the perturbation of arginine, glutamate, and putrescine may be related to potential mechanisms of stress resistance in organisms. In addition, it has been demonstrated that glutamine can not only ameliorate oxidative stress, but can also support anti-apoptotic signaling pathways to attenuate tissue damage [62]. The increase in intracellular L-glutamine levels after the addition of NC may be related to the antioxidant effects of NC [63], which also supports the reduction in the apoptosis of NC-treated cells. The significant downregulation of creatine and phosphocreatine after the NC pretreatment prevented the depletion of ATP stores due to oxidative glycolysis caused by hypoxia, which is beneficial for energy regulation and the protection of cellular functions [51]. In conclusion, NC may exert antioxidant activity, regulate energy metabolism to resist hypoxic stress, and reduce apoptosis.

## 5. Conclusions

In summary, nitidine chloride has a protective effect on hypoxic mitochondria, which is mainly achieved by alleviating oxidative stress and inhibiting apoptosis and mitochondrial autophagy mediated by PINK1-Parkin. In addition, in response to hypoxic stress, nitidine chloride also regulates aminoacyl tRNA biosynthesis; arginine and proline metabolism; glycine, serine, and threonine metabolism; phenylalanine, tyrosine, and tryptophan biosynthesis; and phenylalanine metabolism. This provides a reference for the application of nitidine chloride to regulate the mammary lactation function of milk buffalo.

## Figures and Tables

**Table 1:** Differentially expressed metabolites between groups.

Group	MS2 Name	VIP	*p*-Value	Log_2_ (Fold Change)
Hypoxia group vs. control group	Glycine	2.06503945	0.0115174	1.56481073
	L-Proline	1.8146664	0.0057282	0.27900371
	L-Valine	1.94696133	0.01744092	0.31340213
	L-Phenylalanine	1.82832093	0.01943034	0.42218272
	L-Leucine	1.97497522	0.00369183	0.47928603
	L-Tyrosine	2.14542951	0.00292428	0.59032371
	L-Arginine	2.08233247	0.019683	6.06119033
	L-Threonine	1.82951319	0.00912492	0.36857934
	L-Lysine	1.77506075	0.02069434	0.51409962
	L-Serine	1.68919665	0.0159576	0.34383581
	L-Tryptophan	1.99323675	0.00311541	0.37794000
	D-Serine	1.72542243	0.02710168	0.47073115
NC group vs. control group	L-Proline	1.659236081	0.000632453	0.51476555
	L-Valine	1.722235647	0.000137293	0.43199016
	L-Arginine	1.670055128	0.002407597	2.22884474
	L-Serine	1.180211779	0.020463962	−0.49094164
	L-Tryptophan	1.554519661	0.000793316	−0.46717967
	L-Glutamine	1.51173143	0.00077209	0.84978503
	L-Histidine	1.01517057	0.00127704	1.08186686
	Ornithine	1.2597684	0.01981797	−0.24623455
	Creatine	1.1578884	0.00026026	−1.25428645
	Putrescine	1.1294524	0.04979805	0.49235220
	Fumaric acid	1.56449674	0.00158428	−0.94732761
	Phosphocreatine	1.80772302	1.3803 × 10^−9^	−3.88623870
	Sphinganine	1.50844256	0.01995072	3.14467020
	3-Dehydrosphinganine	1.28612153	0.01472151	0.61942296
	Sphingosine	1.48125934	0.01420779	1.04958982
	Dephospho-CoA	1.70834635	5.6155 × 10^−6^	1.50128981
	Uracil	1.17502289	0.03087762	0.60378648
Hypoxia group vs. NC group	L-Proline	1.34323632	0.03605266	−0.23576184
	L-Phenylalanine	1.32179184	0.02690625	0.37615720
	L-Leucine	1.3151479	0.02560117	0.31427600
	L-Tyrosine	1.78363715	0.00051011	0.73299900
	L-Arginine	1.28769964	0.02423434	3.83234558
	L-Threonine	1.88995394	0.00017607	0.50570638
	L-Lysine	1.38158028	0.01888125	0.65851161
	L-Serine	1.64398014	0.0009724	0.83477745
	L-Tryptophan	1.92948048	3.9033 × 10^−5^	0.84511967
	L-Glutamine	1.99459396	0.00057333	−0.76479694
	Creatine	1.36071106	5.3666 × 10^−5^	1.38760715
	Putrescine	1.77582442	0.00121064	−1.04116564
	Phosphocreatine	1.98055947	0.00138537	3.85284781
	D-Serine	1.41171874	0.00897215	0.73022943
	Pyruvic acid	1.46697958	0.01852634	1.30657351

## Data Availability

The original contributions presented in the study are included in the article; further inquiries can be directed to the corresponding author.

## Supplementary Materials

The following supporting information can be downloaded at: https://www.mdpi.com/article/10.3390/ani14203016/s1. Table S1: Primary antibodies used for immunofluorescence and Western Blot; Table S2. Secondary antibodies used for immunofluorescence and Western Blot.
