# Supplementary material for: Nitidine Chloride Alleviates Hypoxic Stress via PINK1-Parkin-Mediated Mitophagy in the Mammary Epithelial Cells of Milk Buffalo
Source: Animals (Basel). 2024 Oct 18;14(20):3016. doi: 10.3390/ani14203016 (PMC11505235; doi:10.3390/ani14203016)
Supplement: Supplementary file 1 [file animals-14-03016-s001.zip › Supplementary Tables.pdf]

Table S1. Primary antibodies used for immunofluorescence and Western Blot

Antibody	Company	Cat. Number	Host species	Dilution
LC3	Abcam [1]	ab128025	Rabbit	1:50
TOM20	Proteintech [2]	66777-1-Ig	Mouse	1:50
β-actin	Proteintech	66009-1-Ig	Mouse	1:5000
Drp1	Proteintech	12957-1-AP	Rabbit	1:5000
VDAC1	Proteintech	55259-1-AP	Rabbit	1:3000
Parkin	Proteintech	66674-1-Ig	Mouse	1:4000
PINK1	Proteintech	23274-1-AP	Rabbit	1:1000
Mfn1	Proteintech	13798-1-AP	Rabbit	1:1000
Mfn2	Proteintech	12186-1-AP	Rabbit	1:1000
LC3B	Proteintech	14600-1-AP	Rabbit	1:1000

[1] Abcam, Cambridge, UK
[2] Proteintech, Chicago, IL, USA

Table S2. Secondary antibodies used for immunofluorescence and Western Blot

Antibody	Company	Cat. Number	Host species	Dilution
Goat Anti-Rabbit IgG (H+L) Secondary Antibody (Alexa Fluor488)	Abiowell [1]	AWS00054b	Goat	1:200
Goat Anti-Mouse IgG (H+L) Secondary Antibody (Alexa Fluor594)	Abiowell	AWS0004b	Goat	1:200
Goat anti-Mouse IgG (H+L) Secondary Antibody, HRP	Abiowell	AWS0001	Goat	1:5000
Goat anti-Rabbit IgG (H+L) Secondary Antibody, HRP	Abiowell	AWS0002	Goat	1:5000

[1] Abiowell, Changsha, China
