# Supplementary figures and images for: Nitidine Chloride Alleviates Hypoxic Stress via PINK1-Parkin-Mediated Mitophagy in the Mammary Epithelial Cells of Milk Buffalo
Source: Animals (Basel). 2024 Oct 18;14(20):3016. doi: 10.3390/ani14203016 (PMC11505235; doi:10.3390/ani14203016)

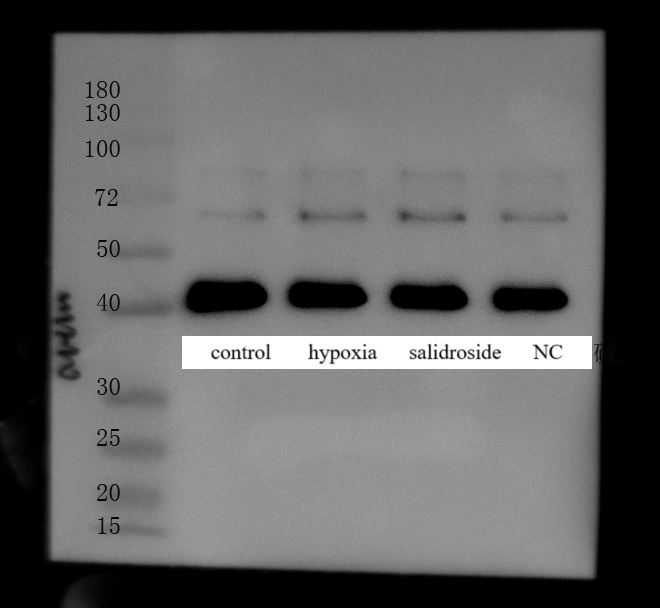

Supplement: Supplementary file 1 [file animals-14-03016-s001.zip › raw data for WB/actin.tif]

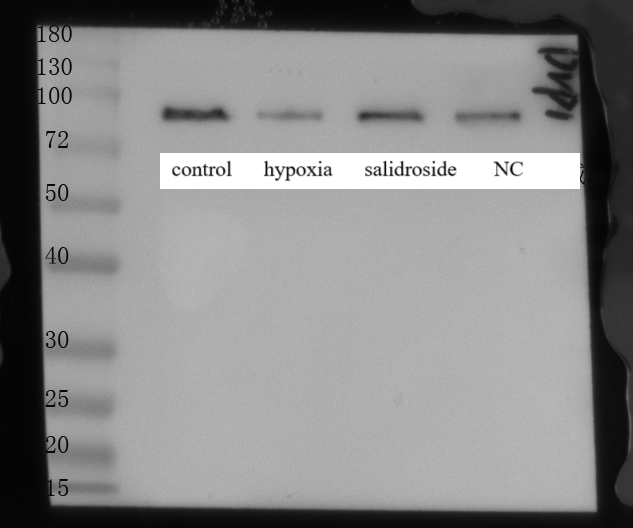

Supplement: Supplementary file 1 [file animals-14-03016-s001.zip › raw data for WB/Drp1.tif]

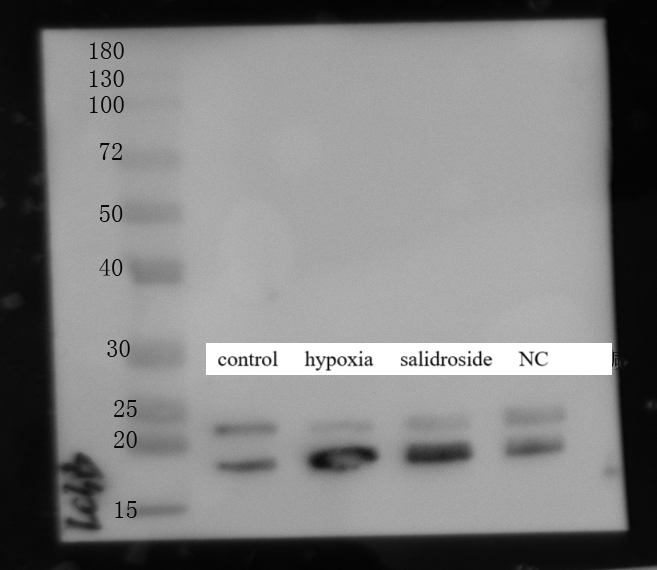

Supplement: Supplementary file 1 [file animals-14-03016-s001.zip › raw data for WB/LC3B.tif]

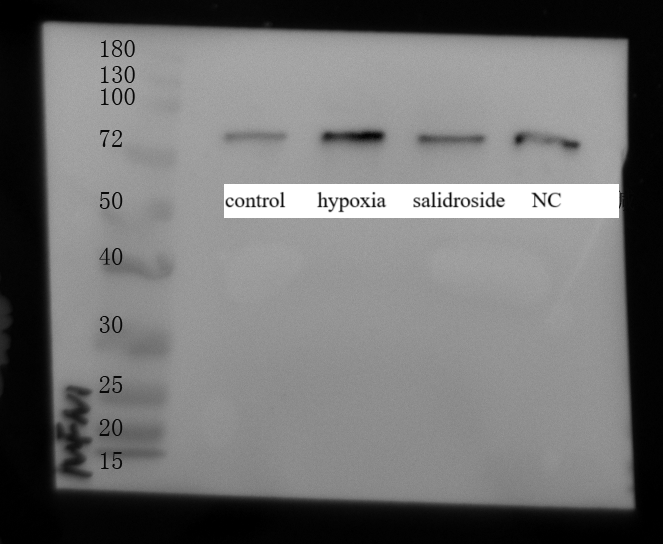

Supplement: Supplementary file 1 [file animals-14-03016-s001.zip › raw data for WB/MFN1.tif]

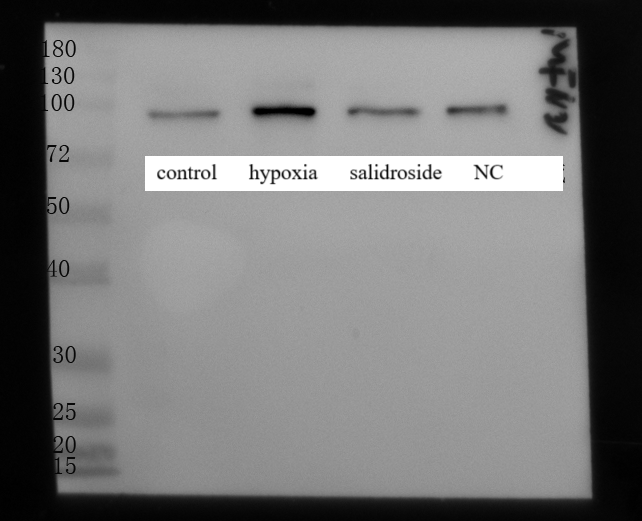

Supplement: Supplementary file 1 [file animals-14-03016-s001.zip › raw data for WB/MFN2.tif]

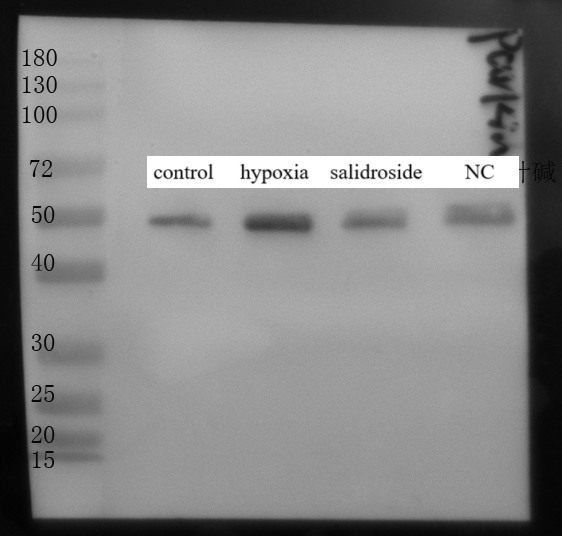

Supplement: Supplementary file 1 [file animals-14-03016-s001.zip › raw data for WB/Parkin.tif]

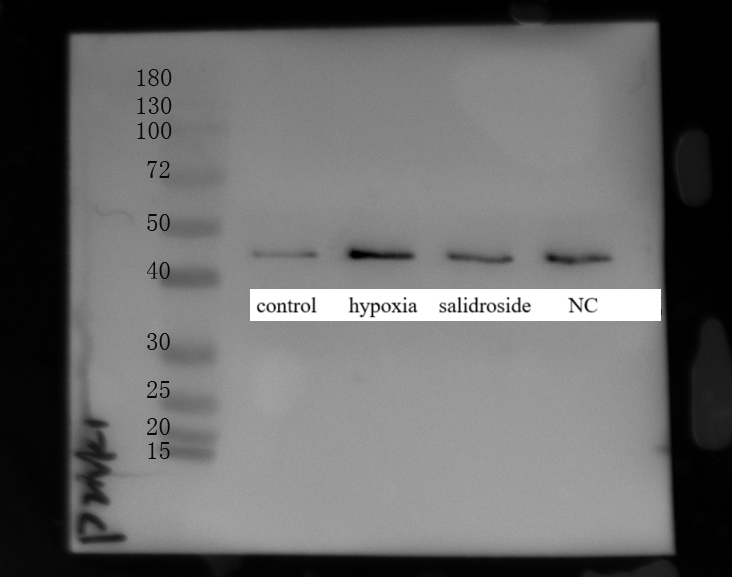

Supplement: Supplementary file 1 [file animals-14-03016-s001.zip › raw data for WB/PINK1.tif]

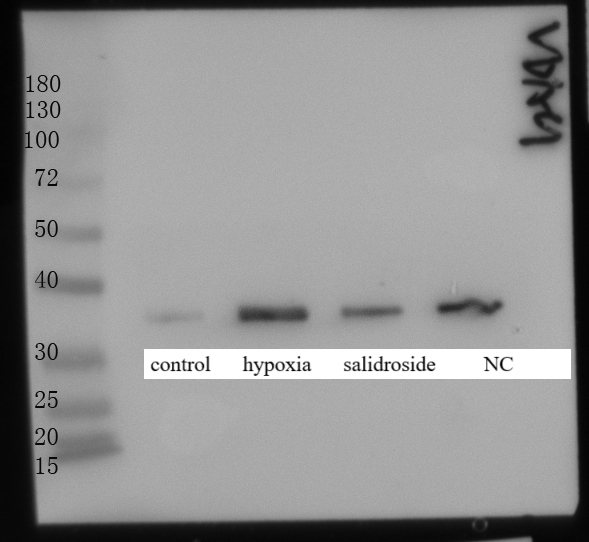

Supplement: Supplementary file 1 [file animals-14-03016-s001.zip › raw data for WB/VDAC1.tif]
